# The amphibian microbiome exhibits poor resilience following pathogen-induced disturbance

**DOI:** 10.1038/s41396-020-00875-w

**Published:** 2021-02-09

**Authors:** Andrea J. Jani, Jessie Bushell, Cédric G. Arisdakessian, Mahdi Belcaid, Daniel M. Boiano, Cathy Brown, Roland A. Knapp

**Affiliations:** 1 Department of Oceanography, Center for Microbial Oceanography: Research and Education (CMORE), University of Hawai’i at Mànoa, Honolulu, HI USA; 2 Pacific Biosciences Research Center, University of Hawai’i at Mànoa, Honolulu, HI USA; 3 San Francisco Zoological Society, San Francisco, CA USA; 4 Information and Computer Sciences, University of Hawai’i at Mànoa, Honolulu, HI USA; 5 Sequoia and Kings Canyon National Parks, National Park Service, Three Rivers, CA USA; 6 USDA Forest Service, Stanislaus National Forest, Sonora, CA USA; 7 Sierra Nevada Aquatic Research Laboratory, University of California, Mammoth Lakes, CA USA

**Keywords:** Microbial ecology, Community ecology

## Abstract

Infectious pathogens can disrupt the microbiome in addition to directly affecting the host. Impacts of disease may be dependent on the ability of the microbiome to recover from such disturbance, yet remarkably little is known about microbiome recovery after disease, particularly in nonhuman animals. We assessed the resilience of the amphibian skin microbial community after disturbance by the pathogen, *Batrachochytrium dendrobatidis* (Bd). Skin microbial communities of laboratory-reared mountain yellow-legged frogs were tracked through three experimental phases: prior to Bd infection, after Bd infection (disturbance), and after clearing Bd infection (recovery period). Bd infection disturbed microbiome composition and altered the relative abundances of several dominant bacterial taxa. After Bd infection, frogs were treated with an antifungal drug that cleared Bd infection, but this did not lead to recovery of microbiome composition (measured as Unifrac distance) or relative abundances of dominant bacterial groups. These results indicate that Bd infection can lead to an alternate stable state in the microbiome of sensitive amphibians, or that microbiome recovery is extremely slow—in either case resilience is low. Furthermore, antifungal treatment and clearance of Bd infection had the additional effect of reducing microbial community variability, which we hypothesize results from similarity across frogs in the taxa that colonize community vacancies resulting from the removal of Bd. Our results indicate that the skin microbiota of mountain yellow-legged frogs has low resilience following Bd-induced disturbance and is further altered by the process of clearing Bd infection, which may have implications for the conservation of this endangered amphibian.

## Introduction

Ecological communities exist in dynamic environmental landscapes, and experience changes ranging from regular incremental shifts such as light and nutrient gradients to large scale disturbances such as disease outbreaks or storm events. Disturbance can alter the structure of ecological communities: it can maintain long term community diversity [1], but can also degrade community or ecosystem function [2–4]. Understanding what enables a community to maintain stable structure or function in the face of disturbances is a fundamental aim of ecology, and has increasing practical importance as human activities alter the frequency and intensity of disturbances such as fire, storms, drought, and disease outbreaks [5].

Community stability comprises resistance and resilience. Resistance is the capacity for a community to resist change due to disturbance. In contrast, resilience is the rate at which a community recovers to its initial state after a disturbance has occurred, and is measured as either the degree of recovery at a given time point or the time required for complete recovery [6]. A growing number of studies has examined resistance (or its converse, sensitivity) to disturbance, but far fewer studies have explicitly tested resilience [6]. Studies so far have found that resilience often varies among study systems. For example, soil communities often fail to return to baseline structure [7], while lake microbial communities showed complete recovery [8, 9]. Among host-associated microbial communities, resilience has arguably been most studied in humans, particularly the human gut. The human gut microbiome showed partial recovery following a variety of perturbations, including antibiotics [10, 11], severe diarrhea [12], and diet interventions [13]. Studies of microbiome resilience in nonhuman animals are rare [6], but see [14, 15].

Exposure of a host to an infectious pathogen can represent a disturbance to the microbiome [16–18]. However, little is known about the resilience (recovery) of nonhuman animal microbiomes following disturbance by infectious disease. *Batrachochytrium dendrobatidis* (Bd) is a chytrid fungus that infects the skin of amphibians and causes the potentially lethal disease chytridiomycosis [19]. Since its discovery just over two decades ago, Bd has emerged as a global threat to amphibians, affecting hundreds of amphibian species and driving massive population declines [20–23]. The need to control the disease has spurred research into possible treatments, including vaccination, control of reservoir hosts, and probiotics [24–26]. Research on probiotic efficacy has yielded variable results: bacteria isolated from amphibian skin inhibited Bd growth or infection in some cases (e.g., [27, 28], but not others [29, 30]). Understanding the stability of the amphibian skin microbiome is relevant to probiotic success or failure for two reasons. First, Bd infection disturbs the microbiome, indicating that disease mitigation strategies may benefit from the ability to improve microbiome stability [17]. Second, and somewhat paradoxically, microbiome stability may hinder establishment of potentially beneficial probiotics, a phenomenon sometimes referred to as colonization resistance. Understanding of the factors that shape and alter the amphibian microbiome has grown considerably in recent years. Several studies have examined the factors associated with amphibian microbiome composition and diversity (e.g., [31–39]). In addition, studies have experimentally measured effects of disturbances, including Bd or viral infection [17, 40–42] and antibiotic exposure [43], on the microbiome. From these studies, it is clear that Bd infection can disturb the microbiome. However, data on the capacity for the microbiome to recover from such disturbances are extremely rare.

In this study, we assess the recovery, after disturbance by Bd, of amphibian-associated microbial communities (also referred to as microbiota). Our focal host species is the mountain yellow-legged frog (*Rana muscosa*). *Rana muscosa* forms a species complex with *Rana sierrae*, and both are endemic to the Sierra Nevada and severely threatened by Bd [23, 44]. Bd infection disturbs the microbiome of these frogs [17], and as such understanding microbiome resilience in this species is particularly important. In this study, we exposed mountain yellow-legged frogs to Bd (or sham inoculum) and later cleared frogs of infection using an antifungal drug. We present data on Bd infection and microbiome composition and diversity before infection, during the infected stage, and after clearance of the pathogen to test if clearing frogs of Bd leads to recovery of the original microbiome structure.

## Materials and methods

### Ethics statement

Collection and handling of *R. sierrae* and *R. muscosa* was planned so as to minimize impacts to individuals or populations. Captures were part of a conservation effort and not conducted solely for research purposes. Work was conducted under permits from the US Fish and Wildlife Service, National Park Service, and CA Department of Fish and Wildlife, with approval from the UCSB and UC Davis IACUC and the San Francisco Zoo Research Review Committee. See Supporting Materials for permit numbers.

### Study design

This study was part of an effort to restore *R. muscosa* and *R. sierrae* populations through “head-starting” (captive rearing followed by release to the wild). Eighty-seven out of 125 frogs were exposed to Bd in an attempt to immunize against future infection [26], and then cleared of Bd using an antifungal drug, prior to release. A random subset of 38 frogs served as controls and were not exposed to Bd. Skin microbiome swabs were collected from a subset of frogs at three time points: pre-infection (at the start of the experiment, before Bd exposure), post-infection (1 week after Bd-exposure), and recovery period (3 weeks after treating frogs with an antifungal drug to clear Bd infections). Swabs to quantify Bd infection were collected approximately weekly. The study ended 48 days after Bd exposure. The entire study was conducted in the laboratory, before frogs were re-released to the wild.

### Frog populations and handling

Frogs in this study came from the Sierra Nevada mountains, California, USA (Table S1A). Four source populations of *R. muscosa* are located in the southern Sierra (Sequoia and Kings Canyon National Parks), and recently suffered Bd-induced declines. A fifth population (*R. sierrae*, from Plumas National Forest in the northern Sierra) was included in the conservation (head-starting) effort but is excluded from this study because all Plumas frogs were exposed to Bd (there were no control frogs from Plumas) due to conservation priorities. However, for completeness an analysis comparing microbial communities between Plumas and the four *R. muscosa* populations is provided in the supporting Materials.

Frogs were collected as tadpoles or metamorphs using hand nets and transported from field sites to vehicles in 4 l plastic jugs filled with lake water and equipped with aerators (tadpoles) or in individual 60 ml plastic containers containing ~10 ml of lake water (metamorphs). Animals were transported to the San Francisco Zoo, where they were housed in tanks with tap water purified by reverse osmosis and supplemented with Kent Marine R/O Right. Collections took place in the summers of 2015 and 2016. Animals were reared in captivity for 1–2 years to ensure acclimation to the lab and allow all animals to complete metamorphosis prior to beginning the study. (*Rana muscosa* is a long-lived species: in the wild, development from egg through metamorphosis generally takes 2–3 summers (about 1–2 years), and total life spans of 9–12 years are common (Knapp, unpublished). All animals in this study were adults or late-stage sub-adults and were 2–3 years old. Previous research with both *Rana sierrae* and a close relative (*Rana cascadae*) found no difference between the microbiomes of adults and subadults [17, 34]. For this study, frogs were housed in groups of 3–6 individuals per tank, with Bd-infected and uninfected frogs housed in separate rooms as a precaution against cross-contamination. Individual frogs were identifiable by unique passive integrated transponder (PIT) tags. Frogs were fitted with PIT tags either 1 year or 5–6 days prior to the start of the experiment. PIT tag date did not differ between experimental treatment groups (Chi Square *P* = 0.311). Tank water was changed daily.

### Bd exposures (immunizations)

Sierra Nevada Bd isolates were cultured on tryptone agar and Bd inocula and sham inocula were prepared as described in [33], using the same Bd strains. On each of 3 consecutive days, 10^6^ live zoospores (250,000 of each of the four strains), or a sham inoculum (prepared from agar media plates without Bd), was added to each frog tank. Tank water was not changed during the 3-day inoculation period.

### Itraconazole treatment

Three weeks after Bd infection, frogs were treated with itraconazole for 6 days to clear Bd infection [45]. Itraconazole treatment is a common method for clearing amphibians of Bd infection [46], and has been used by our group to treat Bd infection in *R. muscosa*. All frogs (infected and uninfected) were treated with itraconazole to control for any side effects of the drug.

### Swab collection and processing

Skin-associated microbes were collected using sterile synthetic swabs as described previously [33]. Frogs were rinsed with sterile water prior to swabbing. Bd swabs were collected 1 day prior to Bd exposure and approximately weekly for the duration of the experiment (48 days). Bd swabs were collected from all frogs (*N* = 125). Microbiome swabs were collected from a subset of 59 frogs (41 Bd-exposed, 18 unexposed) on three dates: immediately before Bd exposure, 1 week after Bd exposure, and 3 weeks after antifungal treatment (7 weeks after Bd exposure). When swabbing any given frog, we always collected the microbiome swab first and the Bd swab second because pilot data showed no significant difference in Bd loads obtained from first or second swabs (Jani, unpublished). DNA was extracted from swabs, including negative controls, using the Prepman Ultra reagent (Applied Biosystems) as described previously [47].

### Quantification of Bd infection

Bd infection was quantified from swab DNA by quantitative PCR (qPCR) using a probe-based (Taq-Man, Applied Biosystems) assay that targets the internally transcribed spacer (ITS) region, with conditions, primers, and probe sequences following [48]. A 5-point standard curve was included on each plate (range of 10^2^–10^6^ ITS copies). Bd load data are in units of ITS gene copies per swab, and are log10-transformed.

### Sequencing library preparation

We prepared a multiplexed library for Illumina sequencing following Kozich [49]. Briefly, indexed oligonucleotides 341F (CCTACGGGNGGCWGCAG) and 785R (GACTACHVGGGTATCTAATCC) were used to amplify the V3-4 region of the 16S rRNA gene [50]. PCR products were purified using a PCR Purification and Normalization kit (Charm Biotech), then pooled in equimolar quantities. The library was sequenced on one lane of an Illumina MiSeq with v3 chemistry and 300 paired end cycles.

### Bioinformatic processing of Illumina sequence reads

We pre-processed raw sequence data into exact (amplicon) sequence variants (ASVs) at the 100% nucleotide identity using the dada2 R package [51]. Reads were truncated at position 260/190 (forward/reverse read). Reads were discarded using the filterAndTrim() function if they contained one or more bases with quality scores <2, or more than 3 expected errors. Denoising was performed with the learnError() and dada() functions with default parameters. Reads were merged using the mergePairs() function, and any pairs with an overlap of fewer than 20 bases, or with more than one mismatch, were discarded. We used mothur v1.42.3 [52] and the Silva (release 132) database [53] to align and annotate sequences. Sequences with a start or stop position outside the 5–95th percentile range (over all sequences) were discarded. We removed potential chimeras with chimera.vsearch(). Taxonomies were assigned using classify.seqs() and classify.otus(). We removed all mitochondrial or chloroplast ASVs, as well as sequences with no annotations at the domain level. We standardized the number of sequences across all samples by subsampling 5000 reads per sample using sub.sample(). Samples fewer than 5000 reads were discarded. ASVs were defined as unique “amplicon sequence variants” by dada2; we used the lulu R package [54] to refine ASVs as follows: we merged ASVs if all of the three following conditions were satisfied: (1) They co-occur in every sample, (2) One of the two ASVs has a lower abundance than the other in every sample and (3) they share a sequence similarity of at least 97%. Finally, we discarded ASVs with a total abundance of two or fewer reads. Pipeline code is available from [55]. Relative abundance data were arc-sine(square root) transformed. Table S1B shows the final number of samples in each treatment and time point after bioinformatic quality control.

### Microbiota analysis: overview

For clarity, we refer to the treatment groups that were exposed or not exposed to Bd as “Bd+” and “Bd−”, respectively. Note that the Bd+ treatment is referred to as Bd+ even before frogs were exposed to Bd. We use the terms *pre-infection*, *post-infection*, and *recovery period* to indicate the time periods (phases of the experiment) before frogs were exposed to Bd (or to sham inoculum), after frogs were exposed to Bd, and after itraconazole treatment, respectively.

### Measuring microbial community disturbance

Community change in response to disturbance can be measured in terms of change from an initial baseline. In this study we observed large changes in the microbiota over time, even in control frogs. To tease apart change due to Bd infection from temporal variation due to unknown causes, we measured Bd-induced microbial community shift as the difference between treatment and control frogs at a given time point. We determined the effect of Bd infection and Bd-clearance on microbiome composition, multivariate dispersion, and diversity as follows:

### Composition

We measured turnover in microbial community composition (beta diversity) using weighted Unifrac distances [56]. We used NMDS ordination to display whole-community similarity among samples. To test if community composition differed among groups, we ran permutational multivariate analysis of variance (PERMANOVA, [57, 58]) using the adonis function (Vegan package) in R, with 999 permutations. We first ran PERMANOVA on all data together, with Bd treatment (Bd+ or Bd−), frog source population, frog tank, time period, PIT tag group, and the treatment*time and population*time interactions as predictors. We then ran the PERMANOVA model (without time as a predictor) separately for each time phase of the experiment because we are primarily interested in differences between treatments within each time point.

We used LEfSe [59] to identify bacterial taxa that respond to Bd infection or Bd clearance. LEfSe analyses were done separately for each time point, with Bd treatment as class, source population as subclass, standardized abundances, restriction of pairwise comparisons to within subclass, and defaults for all other settings. We also explored whether taxa affected (or unaffected) by Bd tend to be numerically dominant by identifying ASVs that were both widespread (present in both treatments, at all time points) and abundant (cumulatively accounting for over 90% of all sequence reads). We refer to this set of ASVs as “core” taxa for brevity, noting that the thresholds are arbitrary and not intended to suggest a functional core.

### Dispersion

To test for differences in multivariate dispersion (i.e., among-frog variability) of community composition, we used PermDISP (betadisper function in the Vegan R package), followed by the permutest function to determine statistical significance. PermDISP was performed separately for each time point (comparing Bd+ to Bd−) as well as for all time points combined. Frog population was not included in dispersion analysis because the test does not accommodate multiple predictor variables.

### Alpha diversity

We tested for treatment effects on alpha diversity using linear mixed effects models with Bd treatment, frog population, time point, PIT tag group, treatment*time and population*time as predictors, tank as a random effect, and diversity metrics (observed richness, Chao’s richness, Shannon diversity, and Shannon evenness) as response variables. As for community composition analyses, we first ran the model on all data together, and followed with a separate analysis for each time point of the experiment.

### Visualizing disturbance

Resistance and resilience can be visually represented by plotting the magnitude of disturbance through time (e.g., Shade et al. 2012, [6]). Following this framework, we plotted the distance of the Bd+ frog microbiomes relative to Bd- controls at each time point. For visualization, the difference between the microbial communities of Bd+ and Bd− frogs was calculated as the mean Unifrac distance between Bd+ and Bd− frogs at each time point. (Details in Supporting Materials.)

### Predictors of stability

We explored potential drivers of microbial community stability by testing if factors that vary at the start of the experiment affect stability later in the experiment. Specifically, we used linear regressions to test if the magnitude of community change through time (measured as Unifrac distance) for each frog is affected by initial alpha diversity or PIT tag group. We also asked if individual frogs have inherent differences in microbial community stability by using linear regression to test if stability (magnitude of microbiome shift) during the infection phase predicted stability through the recovery phase. (Details in Supporting Materials.)

### Effects on host physiology

To test if Bd-induced disturbance of the microbiota was linked to changes in frog physiological condition, we measured changes in frog body mass over the course of the study, using two metrics: proportional mass change ([mass_final_-mass_initial_]/mass_initial_) and change in mass adjusted for body length ([mass_final_-mass_initial_]/snout-vent length). We used linear regression to test if mass change was predicted by the magnitude of microbial community shifts (measured as Unifrac distance between two time points for each frog).

## Results

### Microbiome diversity and composition

PERMANOVA of all time points together revealed effects of Bd treatment, population, time period, population*time-period interaction and Bd*time-period interaction (PERMANOVA, *P* < 0.05, Table S2). Tank and PIT tag group had no significant effects. The Bd*time interaction suggests an effect of Bd treatment in some time points but not others, as one might expect given that the “Bd+” group was not yet infected in the pre-infection period. Alpha diversity also varied by time period (*P* < 0.05, Table S3) but not Bd treatment, population, or PIT tag group.

**Fig. 1:**
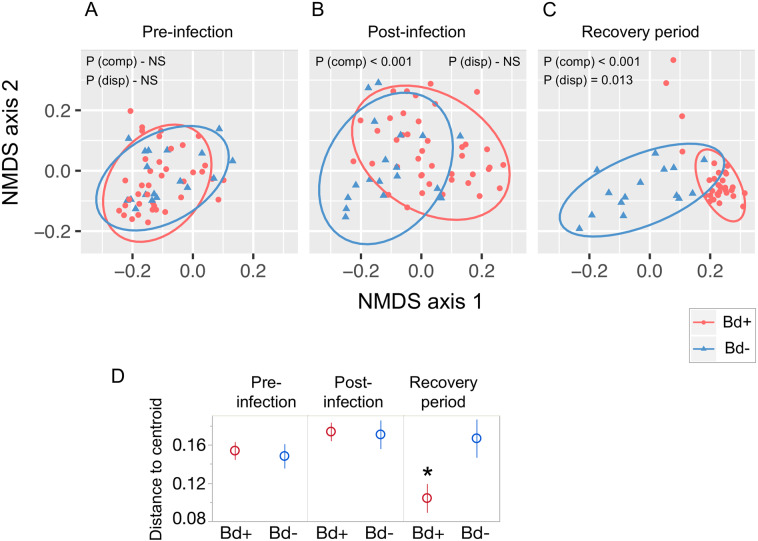
Changes in community composition and dispersion through time. Top panel shows NMDS ordination of microbial communities: **A** Pre-infection, no difference between treatments. **B** Post-infection: composition (position of ellipses) but not dispersion (sizes of ellipses) differed between treatments. **C** Recovery period: Clearing Bd infection did not lead to microbial community recovery. Bd-clearance homogenized the microbiota of Bd-infected (but not uninfected) frogs. Ellipses are 95% normal data ellipses. **D** Microbial community dispersion: mean Unifrac distance (within each treatment-by-time group) of individual microbial communities to the group centroid. Asterisk indicates a group that differs significantly from all other groups (*P* values: Table 5). Error bars are standard error. NMDS was performed on all data together, then separated by experiment phase (**A**, **B**, **C**) for clarity. *P* values correspond to PERMANOVA (comp) and PERMDISP (disp) tests. Ordination stress: 0.06.

We then analyzed each time point separately to focus on between treatment differences within each time point. At the start of the experiment (pre-infection), there were no differences between treatment groups in any metrics of microbiome structure (*P* > 0.05 for tests on alpha diversity, community composition, and multivariate dispersion; Fig. 1A, Tables S4 through S7). Raw relative abundances of common bacterial taxa, for every frog at every time point as well as averaged by time point and treatment, are shown in Figs. S1 and S2.

Bd infection altered microbiome composition on frogs: post-infection, the microbiota of Bd+ and Bd− frogs differed in composition (PERMANOVA, *P* < 0.001, Fig. 1B, Table S6) but not alpha diversity (*P* > 0.05 for all metrics, Table S7) or dispersion (BETADISPER *P* = 0.880, Fig. 1B,Table S4).

**Fig. 2:**
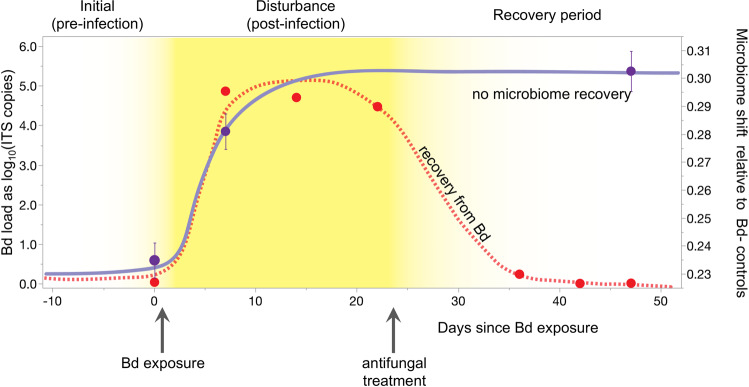
Shifts in Bd infection intensity and microbial community composition through time. Left axis (red symbols): Mean Bd loads through time for Bd+ frogs, showing clearance of Bd after itraconazole treatment. Bd loads are expressed as the number of Bd ITS copies detected per frog, on a log_10_ scale. Right Axis (purple symbols): Shift in microbial communities of Bd+ frogs relative to Bd− controls, measured as mean Unifrac distance between treatment and control. Curved lines are a conceptual diagram following [6], superimposed to visually connect time points for Bd load (red) and microbiome (purple). Error bars indicate standard error. Error bars for Bd load are not visible because they are smaller than size of the circle symbols; See Fig. S3 for box plots of Bd infection loads.

Itraconzole treatment cleared frogs of Bd infection (Fig. 2, Fig. S3) but did not reverse effects of Bd on microbiome composition: microbiota of Bd+ and Bd− treatment groups still differed during the recovery period (PERMANOVA, *P* < 0.001, Fig. 1C, Table S6). In addition, clearing frogs of Bd reduced multivariate dispersion of community composition for Bd+, but not Bd−, frogs. This is demonstrated by a significant effect of Bd treatment on dispersion after Bd clearance (during the recovery period), and not before Bd clearance (Fig. 1C, D. betadisper, *P* = 0.013 for recovery phase, *P* = 0.880 for post-infection). Further analysis of dispersion among all pairwise time*treatment comparisons (pairwise betadisper, all time points included) further showed that the Bd+/recovery phase group had lower dispersion than any other group. Clearing Bd had no effect on alpha diversity (*P* > 0.05 for all metrics, Table S7).

Frog source population had a significant effect on microbiome composition at the post-infection and recovery time points (PERMANOVA, *P* < 0.001, *P* = 0.005, Table S6), but not prior to Bd infection (*P* = 0.145).

For completeness, we reran the above PERMANOVA models with unweighted Unifrac and Bray–Curtis metrics. Results were qualitatively the same as for weighted Unifrac: Bd treatment was not significant pre-infection (unweighted Unifrac *P* = 0.170, Bray–Curtis *P* = 0.411), and was significant post-infection (*P* < 0.001, *P* < 0.001) and during the recovery period (*P* < 0.001, *P* = 0.002).

### Bd infections

Figure 2 shows Bd infection loads over the course of the experiment. All Bd+ frogs became infected by 1 week after being exposed to Bd, with mean Bd load of 67,546 ITS copies per frog). Bd+ frogs remained infected throughout the post-infection period (3 weeks). Of the 275 swabs collected from Bd− frogs (prior to infection or from the Bd− treatment group), six returned positive qPCR results. These were deemed to be false positives because all swabs collected at time points before or after the equivocal swabs from Bd− frogs were PCR-negative, and the Bd loads were very low—below 5 gene copies per qPCR reaction. For comparison, a single Bd cell from Sierra Nevada strains is estimated to have ~60 ITS gene copies [60]. Bd load was not affected by PIT tag group or population (Repeated Measures ANOVA, PIT*time interaction: *P* = 0.901, population*time interaction: *P* = 0.056).

### Microbial community displacement through time

**Fig. 3:**
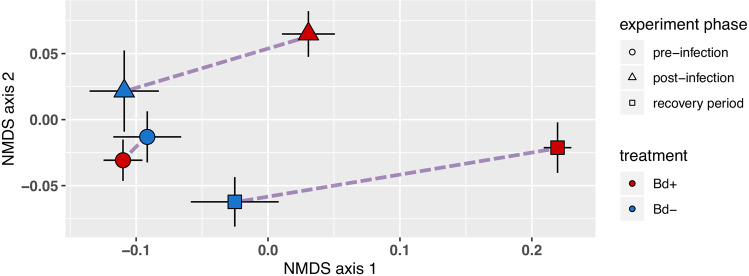
NMDS ordination illustrating microbial community displacement of the Bd+ group relative to the Bd− group in each phase of the experiment. Symbols indicate group mean and standard error of NMDS coordinates from Fig. 1, shown here with all three time points in one plot. Purple dashed lines indicate displacement between treatment and control groups within each time period.

Clearing frogs of Bd infection did not lead to microbial community recovery: the difference between Bd+ and Bd− frogs was not reduced (Figs. 2, 3). The proportion of microbial community variation explained by Bd treatment was greater after Bd clearance than it had been during infection (PERMANOVA, *R*^2^ of Bd effect: 0.12, 0.37 for post-infection and recovery period, respectively).

### Bacterial taxa affected

**Fig. 4:**
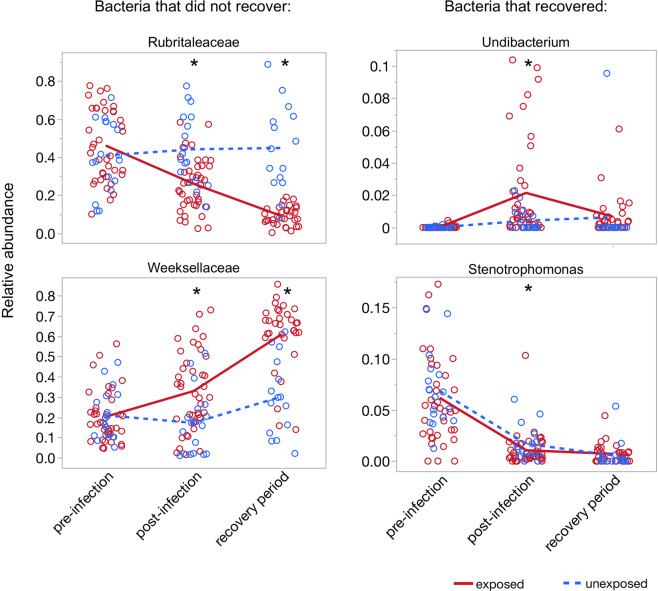
Change in abundance through time for bacterial taxa affected by Bd. Bacterial taxa shown in left panels recovered after Bd clearance, while taxa shown in right panels did not. Plots show raw relative abundance data while statistics are rank-based (LEfSe).

**Fig. 5:**
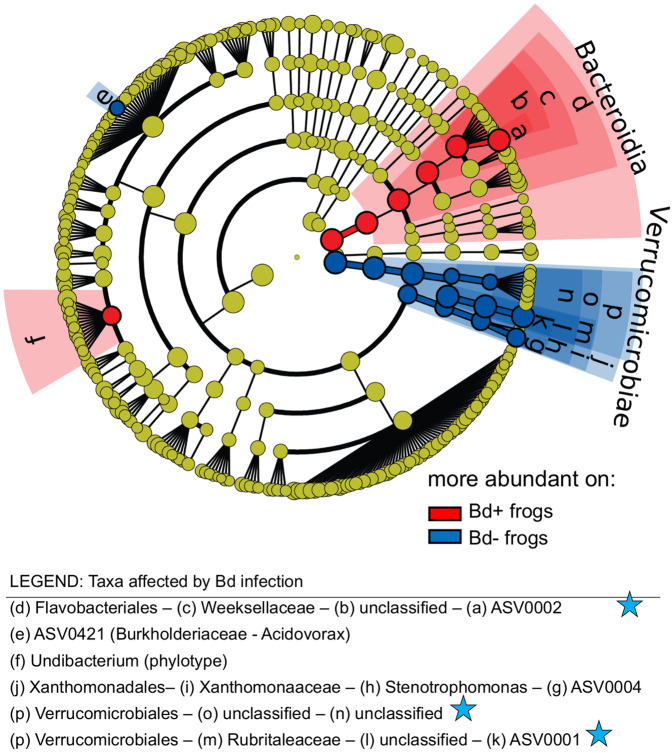
Cladogram of bacterial taxa that differed between Bd-infected and uninfected frogs. Blue stars in legend indicate taxa that remained different after clearance of Bd infections. Cladogram constructed in the program LEfSe.

Bd infection altered the relative abundance of several bacterial taxa (Figs. 4, 5, and S4; Table S8). Figure 4 shows raw relative abundances, but note that LEfSe employs non-parametric rank-based (Kruskal–Wallis) tests, which are less sensitive to the magnitude of differences. LEfSe maintains low false positive rates with the requirement that any difference be consistently detected in all sub-classes (populations) of the data. Bd infection caused the “genus” Undibacterium and an ASV classified to Weeksellaceae as well as the entire family (phylotype) Weeksellaceae to increase relative to controls (Fig. 4). ASVs classified as Rubritaleaceae, Stenotrophomonas, and Verrucomicrobiales declined (Fig. 4). For Stenotrophomonas, the difference between treatment and control was statistically significant but very small. One ASV (ASV421, classified as to Burkholderiaceae) appeared to increase in control (Bd−) frogs, but this may be a spurious finding because ASV421 was very rare (median relative abundance 0 in exposed and 0.0005 in Bd− frogs), which may make the analysis of this ASV sensitive to the limit of detection.

Clearing Bd with itraconazole did not reverse the effects of Bd on the most dominant taxa within the time frame of the experiment, although some less abundant taxa did recover. Weeksellaceae and Rubritaleaceae (both dominant members of the microbiota), and ASVs classified to Verrucomicrobiales did not recover (Fig. 4). Furthermore, Weeksellaceae and Rubritaleaceae continued to increase or decrease, respectively, in Bd+ frogs even after Bd clearance. In contrast, the Undibacterium and Stenotrophomonas appeared to recover after Bd clearance: they no longer differed between treatment groups (Fig. 4). Xanthomonadaceae increased slightly in Bd+ frogs after clearance of Bd infection (Fig. S4). None of the taxa affected by Bd differed prior to Bd infection.

### Core bacterial taxa

Eleven ASVs comprised the “core” microbiome. These included taxa affected by Bd, (Rubritaleaceae, Weeksellaceae, Stenotrophomonas, and an ASV classified as Undibacterium). The remaining core ASVs appeared stable (not significantly affected by Bd infection), and were classified to Pseudomonas (3 ASVs), Gracilibacteria (2), Enterobacteriaceae (1), and Burkholderiaceae (1).

### Predictors of microbial community stability

Microbial community stability, (the magnitude of temporal change, measured as Unifrac distance,) was not predicted by alpha diversity or PIT tag group (linear regression, *P* > 0.05 for all four metrics of alpha diversity; PIT tag group *P* = 0.168). Individual frogs did not appear to have inherent stability levels: stability during the first phase of the experiment (shift from pre- to post-infection) did not predict stability during the second phase (shift from post-infection to recovery period, linear regression, *P* = 0.927).

### Frog physiology

Body mass change was negatively correlated with the shift in the microbiota (Unifrac distance) from pre-infection to post-infection (*P* = 0.010, *P* = 0.023 for proportional mass change and length-adjusted mass change, respectively). When analyzing the Bd+ and Bd− treatments separately, microbiota shift predicted weight loss in the Bd+ group (*P* = 0.005, *P* = 0.016), but not the Bd− group (*P* = 0.474, *P* = 0.097), consistent with the expectation that microbial changes in the Bd− group are due to temporal drift, unassociated with directional changes in host condition.

## Discussion

The maintenance of ecological community function through time depends on community resistance and resilience in the face of disturbances. Although Bd infection has been shown to disturb the microbiome, very little is known about resilience following such disturbance. We found that Bd infection altered the microbiota as expected, but clearing infection did not lead to recovery of the original bacterial community structure. Microbial communities did not regain their original composition, nor did Bd clearance reduce the magnitude of displacement between the microbial communities of Bd-exposed and control frogs. Examining changes in relative abundances of individual bacterial ASVs, we found that the most abundant bacterial groups did not recover (Weeksellaceae and Rubritaleaceae), although some less abundant taxa did appear to recover (Undibacterium and an ASV classified as Stenotrophomonas). These observations suggest that Bd infection may induce a shift to an alternate stable state, such that restoring the host to the uninfected state is insufficient to restore the microbiota to its initial state. Alternatively, recovery may occur but at a rate so slow as to be undetectable in the time frame of our experiment. Our results present an interesting contrast to a study of antiseptic-induced disturbance of the human skin microbiome, which documented marked disturbance followed by complete recovery within 6 h [61]. Both Bd infection and antiseptics represent strong disturbances, but resilience in these studies differed dramatically. The difference in resilience could reflect differences in the disturbance types or differences between human and amphibian skin such as permeability and the presence/absence of mucus glands and hair follicles.

We found that clearing Bd from the skin microbiome reduced among-frog variability in microbiome composition (i.e., reduces dispersion). In other words, clearance of Bd not only failed to reverse Bd-induced changes in *mean* microbiome composition, but also reduced *variability* in composition. The effect on *mean* composition is illustrated by the degree of separation between the blue and red points in the ordination in Fig. 1B and C, while the effect on *variability* in composition is illustrated by the shrinking size of the confidence ellipse around Bd+ individuals in Fig. 1C relative to Fig. 1B. This reduction in variability could be due to either the removal of Bd from the system, or the antifungal drug itself. Direct effects of the drug on the bacterial community are unlikely because itraconazole targets sterols, which are rarely produced by bacteria [62]. In addition, if the drug directly affected bacteria, we would expect to see the effect in all frogs, but we saw it only in Bd+ frogs. However, drug treatment could indirectly affect the microbiome if it were to affect immune cell function, cause side effects in the frogs, or affect resident fungi besides Bd [46, 63]). Symbiotic fungi and bacteria can engage in facultative and inhibitory interactions, either directly or by influencing host immune responses, which could lead to changes in the bacterial community [64, 65]. In this sense, drug treatment could be considered a second disturbance event to which these frogs are normally resistant (since Bd− frogs were not affected), but Bd erodes that resistance. Interestingly, it has been proposed that microbiome disturbance increases variability [66], but we observed the opposite. Increased dispersion might be expected if the disturbance compromises host ability to regulate the microbiome [66]. We hypothesize that, in the current study, reduced variability may instead have been a product of interactions within the microbial community due to the removal of Bd via mechanisms explained below.

Reduced microbial community dispersion might be due to Bd clearance per se, rather than drug treatment. We hypothesize that removing Bd creates vacancies in the microbial community, and that the set of bacteria most likely to occupy those vacancies is similar across frogs, leading to increased similarity among microbial communities. High similarity among frogs in the bacteria that colonize the open space could result from either selection or neutral processes. In the case of selection, certain bacteria might possess traits that allow them to take advantage of resources made available by the removal of Bd, resulting in similar taxa filling the vacancies on most frogs. Alternatively, in a neutral framework, vacancies created by removal of Bd may be filled either through births within the local community (where the local community is the microbiome of one frog) or immigration from the metacommunity species pool, and community differences are not determined by species traits. In the absence of environmental gradients, the metacommunity species pool is assumed to be the same for all frogs. In this case, the sets of bacteria filling vacancies should vary randomly but not differ significantly among frogs, which could reduce among-frog microbiome variability. A previous study of *R. sierrae* indicated that Bd infection may affect immigration and/or drift in a taxon-specific manner [67]. Additional work is needed to determine the mechanism by which Bd clearance reduces microbial community variability. Notably, microbial composition and dispersion exhibited fundamentally different dynamics. Dispersion was unaffected by Bd infection, but strongly affected by Bd clearance. In contrast, composition changed due to Bd infection, and that effect largely persisted after Bd clearance. The ASVs that differed during the recovery period were generally a subset of those affected by Bd infection, suggesting that, in constrast to dispersion, composition did not experience a secondary shift due to Bd clearance.

It is possible that some degree of recovery occurs but was not detectable within the time span or sampling frequency of the experiment [12]. For example, it is possible that the degree of disturbance increased, followed by partial recovery, in the period after our post-infection sample, but this would not be detected without additional post-infection sampling points. However, our post-infection sample was collected on the date on which we measured the highest Bd infection loads (*P* < 0.05, Tukey adjusted), which we expect would limit increases in the magnitude of disturbance beyond our post-infection sampling point, minimizing unobserved recovery. It is also possible that the microbiome recovers very slowly and was therefore not detectable within the time frame of our experiment. While there are few data on amphibian microbiome resilience, one study showed that frog microbiomes disturbed by PIT tagging recovered in 2 weeks or fewer [14]. Another study of disturbance due to a related pathogen, while it did not formally analyze resilience, observed that three newts seemed to recover microbiome composition similar to controls, sometimes <1 week after clearing infection [15]. Furthermore, Loudon et al. [68] showed that transitioning salamanders to new environments resulted in marked shifts in microbiome composition within 1 week. These results suggest that the duration of our experiment would be sufficient to detect some level of resilience.

Captive and wild amphibians exhibit differences in skin microbiome composition [15, 17, 68], and resilience of wild frogs might differ from our observations. For example, it is possible that microbial species found only in natural environmental reservoirs promote resilience. However, studies show that environment does not overwhelm other drivers of microbiome structure: differences in the microbiota among amphibian species and populations persist even under shared environmental conditions [33, 37], several core taxa are found in both lab and field settings [68], and microbial community responses to infection were consistent between lab and field settings [17], suggesting that laboratory studies can provide some insights into processes in nature.

Our study addresses resilience in microbiome structure composition but not function. Our finding that greater community compositional change corresponded to more negative change in body mass for the frogs is consistent with the hypothesis that resilience (return to the initial state) would be beneficial, but without demonstrating that the microbial change *caused* body mass loss, this conclusion is premature. Changes in microbiome structure do not always translate to change in community function, possibly due to functional redundancy among taxa. In addition, functional recovery may proceed at a different rate from taxonomic recovery [69]. Furthermore, disturbance to the microbial community does not necessarily translate to a detriment for the host: change to the microbiota following disease may erode function, but alternatively may indicate selective change toward a microbial community that is more resistant to Bd invasion (i.e., community adaptation to Bd). Studies that measure resistance to Bd during a second exposure (after the recovery period) would be needed to test whether microbiome changes are adaptive. Finally, the microbiome likely serves diverse functions beyond disease resistance, requiring untargeted assays such as functional shotgun metagenomic analyses, but these are rare for the amphibian skin microbiome (but see [70]). Future studies incorporating functional genetic data (e.g., metagenomics), or directly assessing functions of interest are critical for determining the functional significance of Bd-induced disturbances.

Our findings may provide insights for conservation strategies. There are no proven methods for controlling Bd outbreaks in wild populations. Managers and scientists are exploring the release of captive reared frogs, either through captive assurance or head starting programs, to support amphibian populations affected by Bd. These efforts can involve deliberate Bd-exposure and clearance in attempts to immunize frogs prior to release [71]. Our results suggest that management interventions that expose amphibians to Bd could have lasting effects on the microbiota. Further research is needed to determine if this compositional shift is detrimental to microbiome function. If it is, managers may need to weigh the costs and benefits of immunization as part of conservation efforts.

## Supplementary information

SUPPLEMENTAL MATERIAL

## Data Availability

DNA sequence data have been deposited in the NCBI Sequence Read Archive under accession number PRJNA664873.
