# Supplementary material for: The amphibian microbiome exhibits poor resilience following pathogen-induced disturbance
Source: ISME J. 2021 Feb 9;15(6):1628–40. doi: 10.1038/s41396-020-00875-w (PMC8163836; doi:10.1038/s41396-020-00875-w)
Supplement: Supplementary file 1 — SUPPLEMENTAL MATERIAL [file 41396_2020_875_MOESM1_ESM.pdf]

SUPPORTING MATERIALS

PERMIT NUMBERS FOR HANDLING OF RANA MUSCOSA: US Fish and Wildlife Service (TE-40090B-1, TE40087B-2, TE-92167B, biological opinion 08ESMF00-2016-F-1174), National Park Service (SEKI-2015-SCI-0062, SEKI-2016-SCI-0038), CA Department of Fish and Wildlife Scientific Collecting Permit SC-000304. Approval from the UCSB and UC Davis IACUC (UCSB protocol #478, UC Davis #20629, #19974).

VISUALIZING MICROBIOME DISTURBANCE.
Note: Data analyses of community composition were conducted directly on the Unifrac distance matrices. The following metrics were used for visualization purposes only, to enable display of the overall shift in community composition of the Bd-exposed group relative to the control group. We computed the weighted Unifrac distance between each frog in the Bd+ group and each frog in the Bd- group, then took the mean of all comparisons in a given time point. We estimated standard error by dividing the standard deviation by the square root of the number of frogs in the Bd- group. (Although standard error is normally calculated based on the total samples size, in this case the sum of frogs in both treatments, in our case using the number of frogs from both groups treatments would have under-estimated standard error because our questions relate to pairs of frogs in the two groups. We chose the sample size of the Bd- group because there were fewer control frogs than Bd+ frogs, so the Bd- sample size is more conservative.

PREDICTORS OF MICROBIOME STABILITY.
We tested if alpha diversity or frog individual predict stability, where stability is measured as the magnitude of microbiome change (displacement) through time. As a metric of microbiome displacement through time, we calculated the Unifrac distance between time points for each frog. This difference was calculated for the transition from pre-infection to the post-infection stage (phase 1 of the experiment), and again for the transition from the post-infection stage to the recovery stage (phase 2). Many studies focus on alpha diversity as a potential predictor of stability (Downing et al., 2014; Ives and Carpenter, 2007; Shade, 2017). We therefore tested for a correlation between initial alpha diversity and microbiome displacement. It is also possible that individual frogs inherently vary in the stability of their microbiomes (e.g. (Harrison et al., 2019)). We used linear regression to test if stability is an inherent property of individual frogs by testing if microbiome displacement in the first phase of the experiment predicts displacement in the second phase.

Downing, A.L., Brown, B.L., and Leibold, M.A. (2014). Multiple diversity–stability mechanisms enhance population and community stability in aquatic food webs. Ecology *95*, 173–184.

Harrison, X.A., Price, S.J., Hopkins, K., Leung, W.T.M., Sergeant, C., and Garner, T.W.J. (2019). Diversity-Stability Dynamics of the Amphibian Skin Microbiome and Susceptibility to a Lethal Viral Pathogen. Frontiers in Microbiology *10*.

Ives, A.R., and Carpenter, S.R. (2007). Stability and Diversity of Ecosystems. Science *317*, 58–62.
Shade, A. (2017). Diversity is the question, not the answer. The ISME Journal *11*, 1–6.

PLUMAS (*R. SIERRAE*) POPULATION HARBORS DIFFERENT MICROBIOTA FROM *R. MUSCOSA* POPULATIONS.

For the pre-exposure time point, we conducted additional analyses on the effects of frog source population and frog tank on the microbiome. These analyses include a fifth population (Plumas, N=10), and do not include Bd exposure as a factor because, at this time point, no frogs in the experiment were exposed to Bd. We tested if alpha diversity or community composition differed among groups of frogs from the five source populations. Diversity and composition were analyzed by linear models and PERMANOVA, respectively, as described in the main manuscript. RESULTS. Alpha diversity did not differ among source populations (P>0.05 for all diversity metrics). Community composition did differ among source populations (P<0.001). When Plumas frogs were excluded from the analysis, population no longer had a significant effect on the microbiome (main text), indicating that the population effect was primarily due to the fact that the Plumas frogs differed from other populations. We followed up this main PERMANOVA test with pairwise PERMANOVA using the pairwise.adonis function (pairwiseAdonis package in R) conducted using Bray Curtis distance and Holm method multiple comparisons adjustment. The pairwise test showed that Plumas microbiomes differed from Tyndall, Milestone, and Pinchot (P=0.010, 0.016, 0.010, respectively). All other pairwise comparisons between populations were not significant (P>0.05). The Plumas population (located in the northern Sierra) is geographically distant from the other four populations (southern Sierra). The Plumas population is also a separate species (*Rana sierrae*) compared with the other populations (*Rana muscosa*). Therefore environmental differences or frog genetic differences between the northern and southern regions could explain the difference in microbiomes.

NMDS ordination of frog microbiomes prior to Bd exposure. Frog species designations are RAMU (*Rana muscosa*) and RASI (*Rana sierrae*).

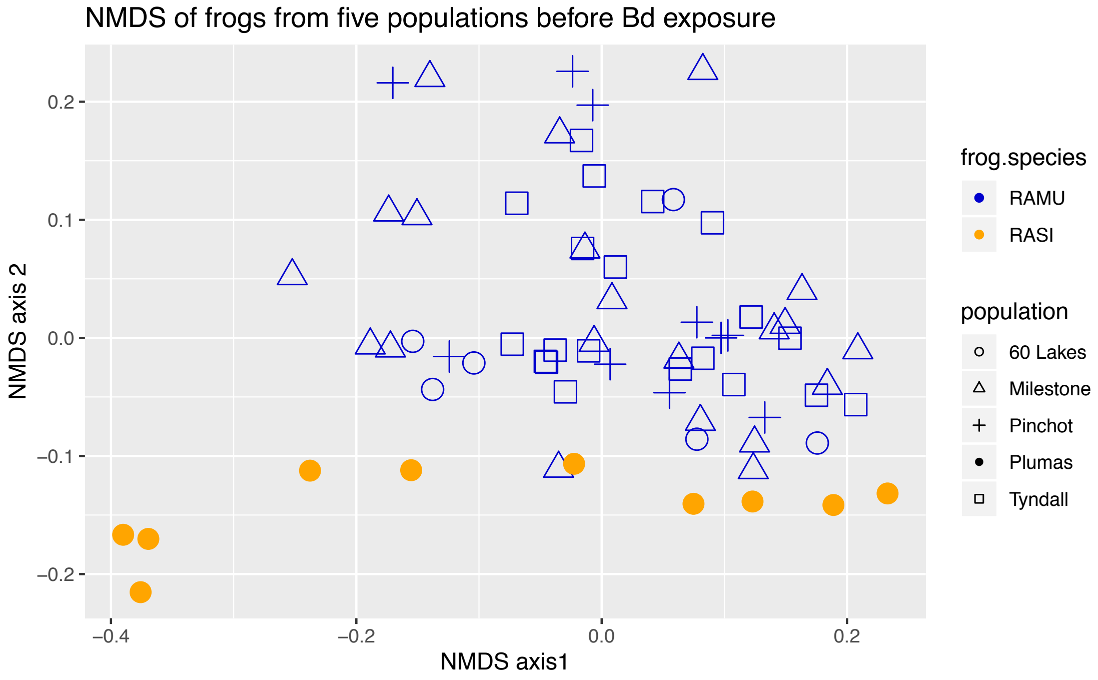

FIGURE S1. Mean relative abundances of common taxa for each treatment and time point.

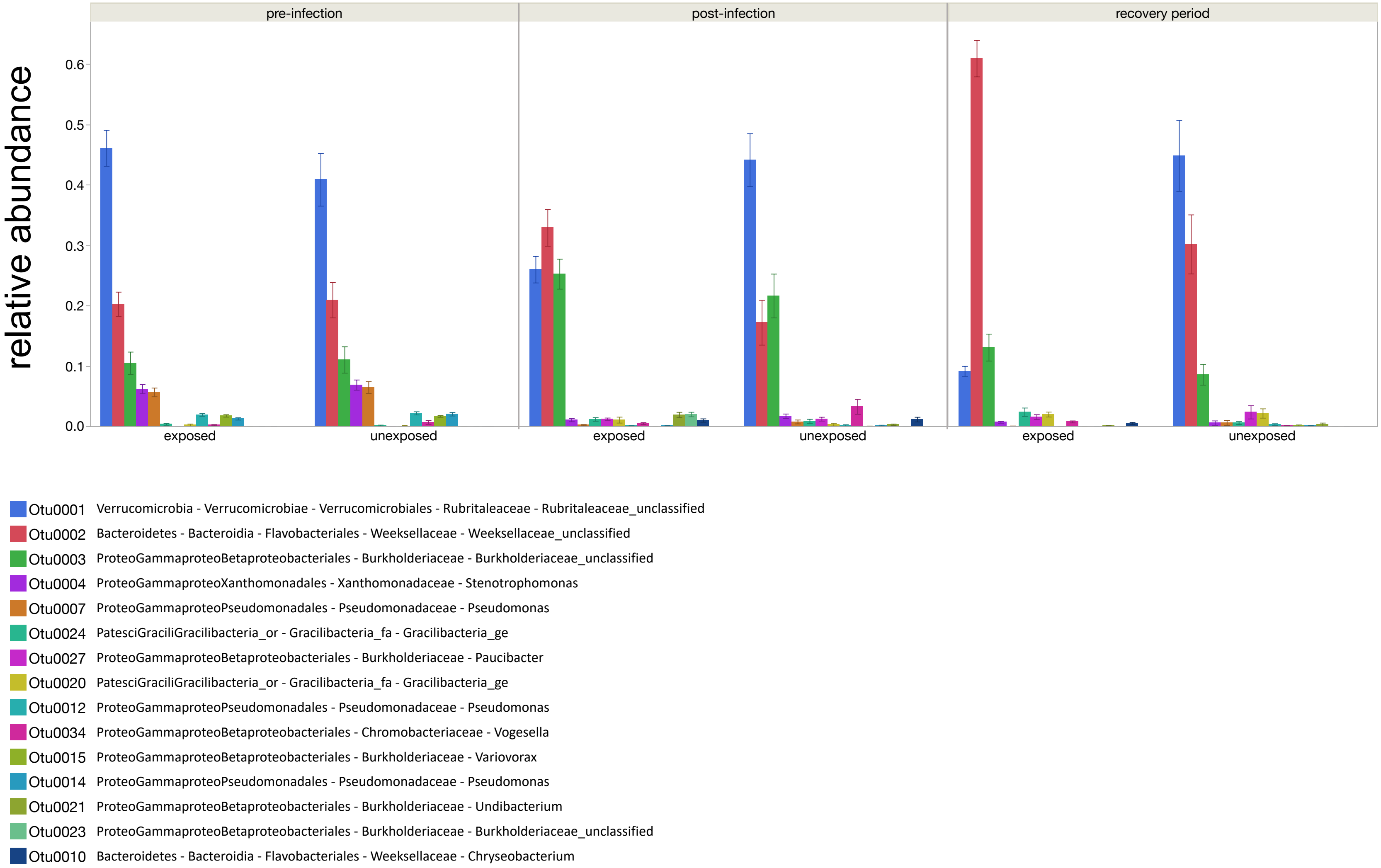

FIGURE S2 Relative abundances of common bacterial taxa on every frog, at every time point. Only ASVs with representing at least 0.1% of reads are shown. Gaps (white space) between tops of stacked bars and 1.0 mark on Y-axis represent rare ASVs. Legend for ASV taxonomy on following page.

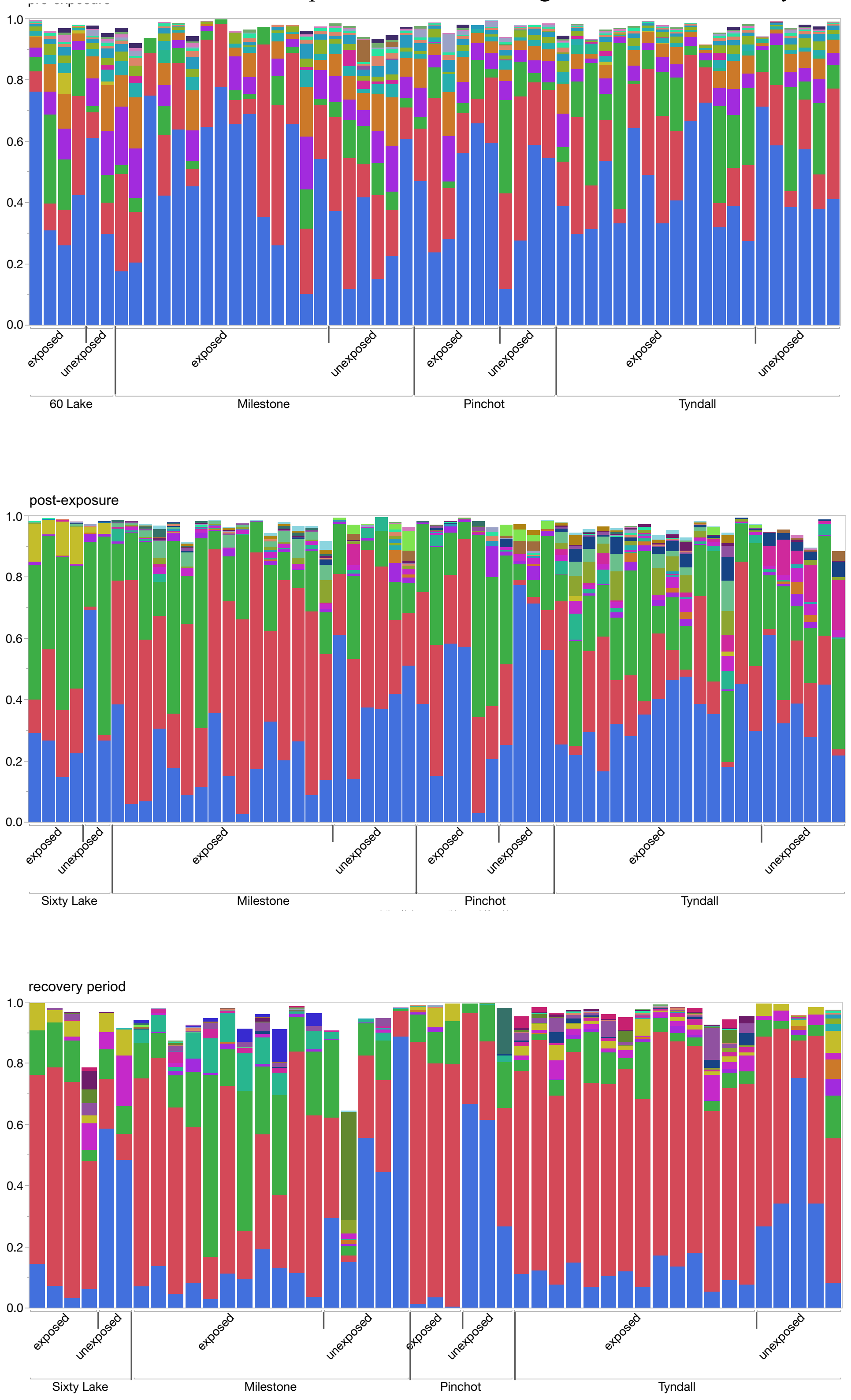

Figure S2 Legend

Otu0001 Verrucomicrobia - Verrucomicrobiae - Verrucomicrobiales - Rubritaleaceae - Rubritaleaceae_unclassified
Otu0002 Bacteroidetes - Bacteroidia - Flavobacteriales - Weeksellaceae - Weeksellaceae_unclassified
Otu0003 ProteoGammaproteoBetaproteobacteriales - Burkholderiaceae - Burkholderiaceae_unclassified
Otu0004 ProteoGammaproteoXanthomonadales - Xanthomonadaceae - Stenotrophomonas
Otu0007 ProteoGammaproteoPseudomonadales - Pseudomonadaceae - Pseudomonas
Otu0024 PatesciGraciliGracilibacteria_or - Gracilibacteria_fa - Gracilibacteria_ge
Otu0027 ProteoGammaproteoBetaproteobacteriales - Burkholderiaceae - Paucibacter
Otu0020 PatesciGraciliGracilibacteria_or - Gracilibacteria_fa - Gracilibacteria_ge
Otu0012 ProteoGammaproteoPseudomonadales - Pseudomonadaceae - Pseudomonas
Otu0034 ProteoGammaproteoBetaproteobacteriales - Chromobacteriaceae - Vogesella
Otu0015 ProteoGammaproteoBetaproteobacteriales - Burkholderiaceae - Variovorax
Otu0014 ProteoGammaproteoPseudomonadales - Pseudomonadaceae - Pseudomonas
Otu0021 ProteoGammaproteoBetaproteobacteriales - Burkholderiaceae - Undibacterium
Otu0023 ProteoGammaproteoBetaproteobacteriales - Burkholderiaceae - Burkholderiaceae_unclassified
Otu0010 Bacteroidetes - Bacteroidia - Flavobacteriales - Weeksellaceae - Chryseobacterium
Otu0033 Bacteroidetes - Bacteroidia - Flavobacteriales - Flavobacteriaceae - Flavobacterium
Otu0025 ProteoGammaproteoEnterobacteriales - Enterobacteriaceae - Enterobacteriaceae_unclassified
Otu0005 Bacteroidetes - Bacteroidia - Sphingobacteriales - env.OPS_17 - env.OPS_17_ge
Otu0031 ProteoGammaproteoEnterobacteriales - Enterobacteriaceae - Enterobacteriaceae_unclassified
Otu0044 ProteoGammaproteoPseudomonadales - Pseudomonadaceae - Pseudomonas
Otu0013 ProteoGammaproteoBetaproteobacteriales - Burkholderiaceae - Delftia
Otu0086 ProteoGammaproteoAeromonadales - Aeromonadaceae - Aeromonas
Otu0037 ProteoGammaproteoBetaproteobacteriales - Chromobacteriaceae - Crenobacter
Otu0016 ProteoGammaproteoPseudomonadales - Pseudomonadaceae - Pseudomonas
Otu0103 ProteoGammaproteoBetaproteobacteriales - Burkholderiaceae - Comamonas
Otu0042 ActinoActinoMicrococcales - Sanguibacteraceae - Sanguibacter
Otu0029 ProteoGammaproteoBetaproteobacteriales - Burkholderiaceae - Rhodoferax
Otu0053 ProteoGammaproteoEnterobacteriales - Enterobacteriaceae - Enterobacteriaceae_unclassified
Otu0008 ProteoGammaproteoBetaproteobacteriales - Methylophilaceae - Methylophilus
Otu0064 ProteoGammaproteoPseudomonadales - Pseudomonadaceae - Pseudomonas
Otu0087 ProteoGammaproteoBetaproteobacteriales - Burkholderiaceae - Acidovorax

FIGURE S3. Bd loads.

Bd loads at each time point for Bd+ (red) and Bd- (blue) treatments. Box plots show median and quantiles; points are outliers. We did not attempt to show every data point (dot plot) because the data distributions are very tight: many points overlap and obscure each other even when jittered. Figure 1 in the main manuscript reflects the same data set, but for clarity shows only means for Bd+ frogs.

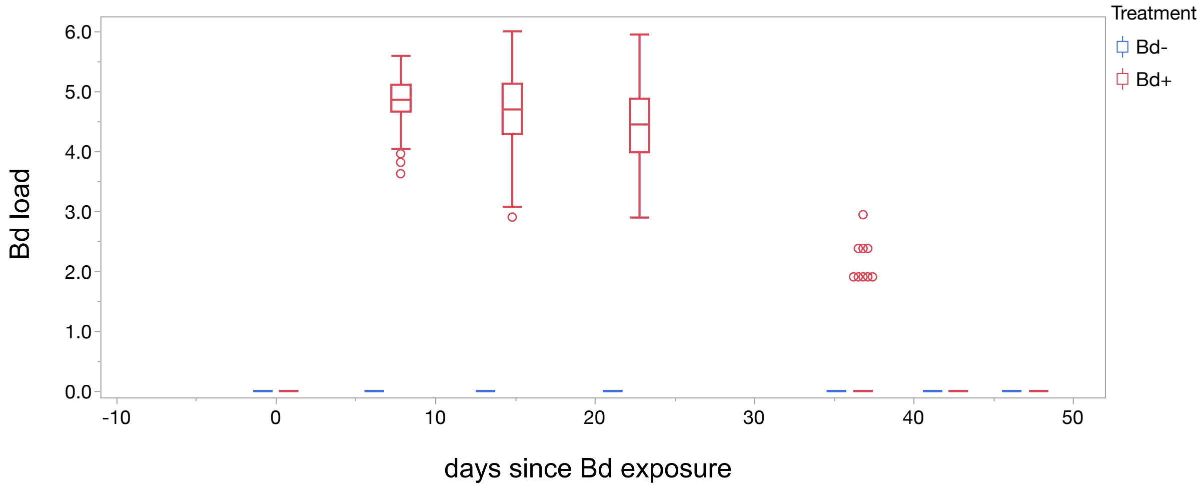

FIGURE S4. TAXA AFFECTED BY BD.
Relative abundances of bacterial taxa affected by Bd exposure. Bar colors distinguish frog source populations. Horizontal black solid and dashed lines show mean and median, respectively, for each frog population. Large plots outlined in red show abundances after Bd exposure (effect of Bd infection). Smaller plots outlined in black show taxa that differed between Bd-exposed and unexposed frogs even after Bd infections were cleared.

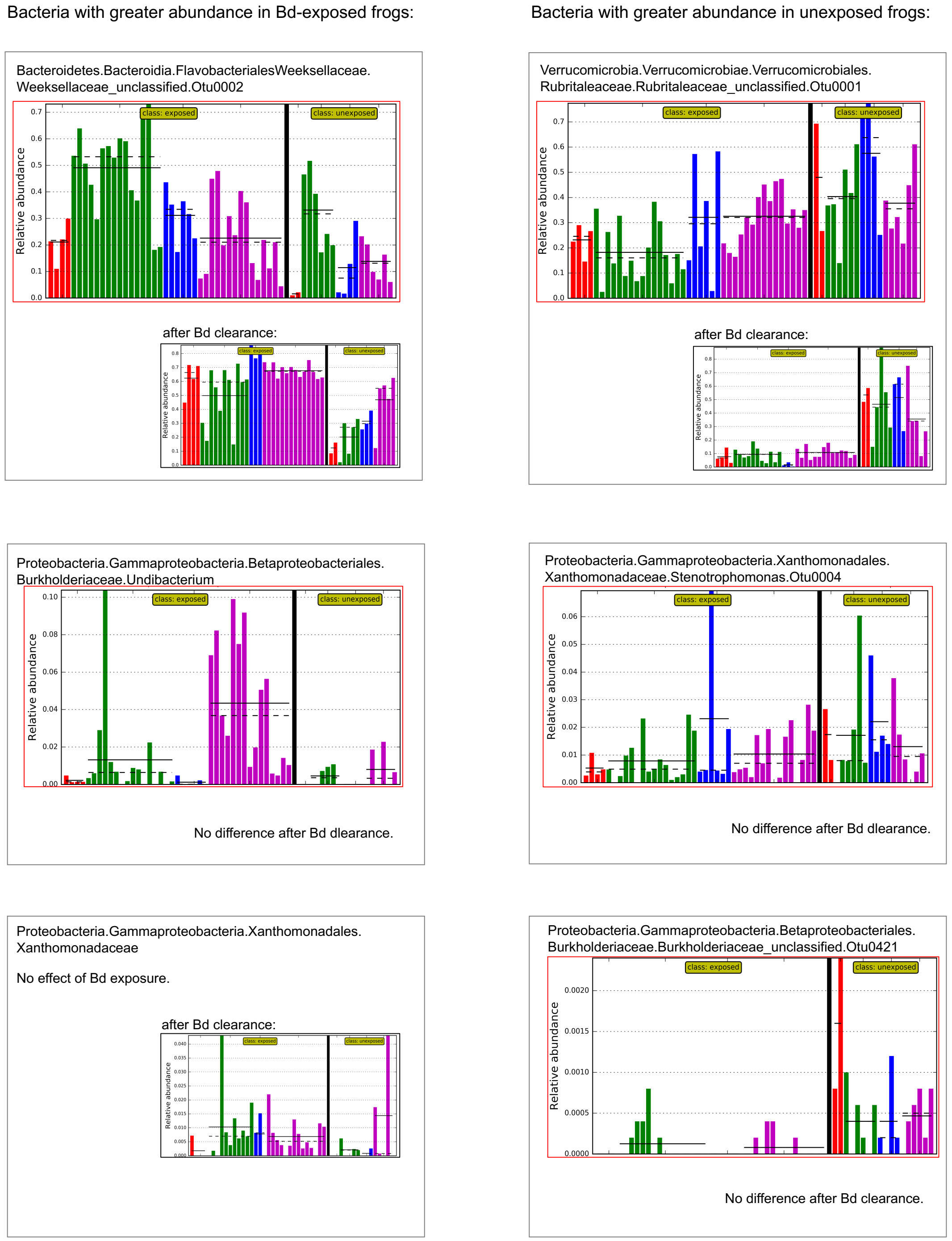

SUPPLEMENTARY TABLES

TABLE S1A,B. SOURCE POPULATIONS AND SAMPLE SIZES.
* Clade refers to genetic clade of the frogs in this study according to (Vredenburg et al., 2007).
** All frogs were swabbed for Bd infection on every sampling date. Microbiome data were generated for a subset of frogs on 3 sampling dates.
Frogs from the Plumas population were excluded from this study because there were no Bd-negative controls for Plumas.

Table S1-A Total sample sizes by population.

population	clade*	# frogs in study**<br>Total and (subset swabbed for microbiome)	exposed	unexposed
Sixty Lake	4	12 (6)	8 (4)	4 (2)
Milestone	4	46 (22)	32 (16)	14 (6)
Pinchot	4	25 (10)	17 (6)	8 (4)
Tyndall	5	42 (21)	30 (15)	12 (6)

Table S1-B. Samples sizes in final analyses (after bioinformatic QC), broken down by treatment and time point.

Population	Pre-infection		Post-infection		Recovery period	
	Exposed	Unexposed	Exposed	Unexposed	Exposed	Unexposed
Sixty Lake	4	2	4	2	4	2
Milestone	15	6	16	6	11	5
Pinchot	6	4	6	4	3	3
Tyndall	14	6	15	6	14	5

Vredenburg, V.T., R. Bingham, Knapp, R., Morgan, J. a. T., Moritz, C., and Wake, D. (2007). Concordant molecular and phenotypic data delineate new taxonomy and conservation priorities for the endangered mountain yellow-legged frog. Journal of Zoology *271*, 361–374.

TABLE S2. MICROBIOME COMPOSITION (PERMANOVA)
FOR ALL TIME POINTS TOGETHER
Effects of experiment phase, Bd treatment, frog population, experimental tank within population, and PIT tag group on microbiome composition. A single PERMANOVA test was run on all three time points together.

term	P-value	R squared
population	<0.001	0.037
experiment phase (i.e., time)	<0.001	0.283
Bd treatment	<0.001	0.063
tank	0.334	NA
PIT tag group	0.601	NA
population x experiment phase	<0.001	0.082
Bd treatment x experiment phase	<0.001	0.057

Table S3. ALPHA DIVERSITY
ANALYSIS WITH ALL TIME POINTS TOGETHER
Linear mixed model:
Y~ Time Period + Bd + Population + Bd*Population + PIT tag group + tank(random)
Data are P-values, Benjamini-Hochberg adjusted.

term	Shannon diversity	"species" (ASVs) observed	Chao's diversity	Shannon evenness
experiment phase (i.e., time)	0.0011	0.0011	0.0078	0.1300
experiment phase x population	0.0432	0.8600	0.8600	0.0259
population	0.8600	0.4423	0.7445	0.8600
Bd treatment x experiment phase	0.8600	0.7715	0.8600	0.8600
Bd treatment x population	0.8600	0.8600	0.8600	0.8600
Bd treatment	0.8600	0.8600	0.8600	0.8600
PIT tag group	0.8600	0.8600	0.8600	0.8600

TABLE S4. DISPERSION, separate test for each time period.
Effects of bd exposure on multivariate dispersion of composition (Betadisper on Unifrac distance)

experiment phase	P-value (Bd exposure)
pre-exposure	0.716
post-exposure	0.880
recovery period	0.013

Table S5. DISPERSION: Pairwise comparisons (across all treatments and time points) of the within-group dispersion. This analysis tests whether any treatment-by-timepoint group has less variable microbiomes (lower within-group dispersion) than other groups.

group 1	group 2	P-value
post-infection, Bd-exposed	recovery period, Bd-exposed	<0.001
pre-infection, Bd-exposed	recovery period, Bd-exposed	0.007
post-infection, unexposed	recovery period, Bd-exposed	0.01
recovery period, Bd-exposed	recovery period, unexposed	0.023
pre-infection, unexposed	recovery period, Bd-exposed	0.049
pre-infection, unexposed	post-infection, Bd-exposed	0.149
pre-infection, Bd-exposed	post-infection, Bd-exposed	0.152
pre-infection, unexposed	post-infection, unexposed	0.255
pre-infection, Bd-exposed	post-infection, unexposed	0.346
pre-infection, unexposed	recovery period, unexposed	0.431
pre-infection, Bd-exposed	recovery period, unexposed	0.537
pre-infection, Bd-exposed	pre-infection, unexposed	0.716
post-infection, Bd-exposed	recovery period, unexposed	0.761
post-infection, Bd-exposed	post-infection, unexposed	0.872
post-infection, unexposed	recovery period, unexposed	0.877

TABLE S6. MICROBIOME COMPOSITION (PERMANOVA) FOR EACH TIME POINT
Effects of Bd treatment, frog population, experimental tank within population, and PIT tag group on microbiome composition, broken down by time point. Bd*population interaction was not significant in any test.

experiment phase	P-value (Bd exposure)	R squared (Bd exposure)	P-value (population)	R squared (population)	P-value (tank)	R squared (tank)	P-value (PIT tag group)	R squared (PIT tag group)
pre-infection	0.442	NA	0.108	NA	0.369	NA	0.165	NA
post-infection	<0.001	0.12	<0.001	0.26	0.002	0.35	0.868	NA
recovery period	<0.001	0.38	0.005	0.12	0.244	NA	0.894	NA

TABLE S7. ALPHA DIVERSITY
SEPARATE ANALYSIS FOR EACH TIME POINT
Linear mixed models run separately for each diversity metric:
Y~ Bd + Population + Bd*Population + PIT tag group + tank(random)
Data are P-values (Benjamini-Hochberg adjusted p-values in parentheses)

**phase**	**term**	**"species" (ASVs) observed**	**Chao's diversity**	**Shannon diversity**	**Shannon evenness**
pre-infection	Bd treatment	0.191 (0.986)	0.255 (0.986)	0.33 (0.986)	0.511 (0.986)
pre-infection	Bd treatment x population	0.265 (0.986)	0.207 (0.986)	0.379 (0.986)	0.539 (0.986)
pre-infection	PIT tag group	0.82 (0.986)	0.614 (0.986)	0.986 (0.986)	0.953 (0.986)
pre-infection	population	0.952 (0.986)	0.985 (0.986)	0.83 (0.986)	0.598 (0.986)
post-infection	Bd treatment	0.833 (0.986)	0.834 (0.986)	0.668 (0.986)	0.648 (0.986)
post-infection	Bd treatment x population	0.292 (0.986)	0.241 (0.986)	0.744 (0.986)	0.221 (0.986)
post-infection	PIT tag group	0.817 (0.986)	0.767 (0.986)	0.747 (0.986)	0.693 (0.986)
post-infection	population	0.087 (0.986)	0.117 (0.986)	0.039 (0.986)	0.076 (0.986)
recovery period	Bd treatment	0.402 (0.986)	0.443 (0.986)	0.528 (0.986)	0.201 (0.986)
recovery period	Bd treatment x population	0.501 (0.986)	0.416 (0.986)	0.731 (0.986)	0.602 (0.986)
recovery period	PIT tag group	0.074 (0.986)	0.095 (0.986)	0.193 (0.986)	0.493 (0.986)
recovery period	population	0.005 (0.233)	0.006 (0.293)	0.026 (0.986)	0.231 (0.986)

TABLE S8. LEfSe RESULTS

Linear discriminant analysis effect sizes (LDA scores) and P values.

Taxonomic group	experiment phase	higher in:	LDA	P
Bacteroidetes	post-exposure	exposed	4.925	0.0021
Bacteroidetes.Bacteroidia	post-exposure	exposed	4.925	0.0021
Bacteroidetes.Bacteroidia.Flavobacteriales	post-exposure	exposed	4.928	0.0021
Bacteroidetes.Bacteroidia.Flavobacteriales.Weeksellaceae	post-exposure	exposed	4.913	0.0025
Bacteroidetes.Bacteroidia.Flavobacteriales.Weeksellaceae.Weeksellaceae_unclassified	post-exposure	exposed	4.919	0.0025
Bacteroidetes.Bacteroidia.Flavobacteriales.Weeksellaceae.Weeksellaceae_unclassified.Otu0002	post-exposure	exposed	4.917	0.0025
Proteobacteria.Gammaproteobacteria.Betaproteobacteriales.Burkholderiaceae.Burkholderiaceae_unclassified.Otu0421	post-exposure	unexposed	3.19	<0.00001
Proteobacteria.Gammaproteobacteria.Betaproteobacteriales.Burkholderiaceae.Undibacterium	post-exposure	exposed	3.947	0.0031
Proteobacteria.Gammaproteobacteria.Xanthomonadales	post-exposure	unexposed	3.505	0.049
Proteobacteria.Gammaproteobacteria.Xanthomonadales.Xanthomonadaceae	post-exposure	unexposed	3.505	0.049
Proteobacteria.Gammaproteobacteria.Xanthomonadales.Xanthomonadaceae.Stenotrophomonas	post-exposure	unexposed	3.505	0.049
Proteobacteria.Gammaproteobacteria.Xanthomonadales.Xanthomonadaceae.Stenotrophomonas.Otu0004	post-exposure	unexposed	3.526	0.037
Verrucomicrobia	post-exposure	unexposed	4.997	0.0009
Verrucomicrobia.Verrucomicrobiae	post-exposure	unexposed	4.997	0.0009
Verrucomicrobia.Verrucomicrobiae.Verrucomicrobiales	post-exposure	unexposed	4.997	0.0009
Verrucomicrobia.Verrucomicrobiae.Verrucomicrobiales.Rubritaleaceae	post-exposure	unexposed	4.995	0.0009
Verrucomicrobia.Verrucomicrobiae.Verrucomicrobiales.Rubritaleaceae.Rubritaleaceae_unclassified	post-exposure	unexposed	4.995	0.0009
Verrucomicrobia.Verrucomicrobiae.Verrucomicrobiales.Rubritaleaceae.Rubritaleaceae_unclassified.Otu0001	post-exposure	unexposed	4.995	0.0009

Verrucomicrobia.Verrucomicrobiae.Verrucomicrobiales.Verrucomicrobiales_unclassified	post-exposure	unexposed	3.495	0.0203
Verrucomicrobia.Verrucomicrobiae.Verrucomicrobiales.Verrucomicrobiales_unclassified.Verrucomicrobiales_unclassified	post-exposure	unexposed	4.02	0.0203
Bacteroidetes	recovery period	exposed	5.191	<0.0001
Bacteroidetes.Bacteroidia	recovery period	exposed	5.191	<0.0001
Bacteroidetes.Bacteroidia.Flavobacteriales	recovery period	exposed	5.223	<0.0001
Bacteroidetes.Bacteroidia.Flavobacteriales.Weeksellaceae	recovery period	exposed	5.201	<0.0001
Bacteroidetes.Bacteroidia.Flavobacteriales.Weeksellaceae.Weeksellaceae_unclassified	recovery period	exposed	5.186	<0.0001
Bacteroidetes.Bacteroidia.Flavobacteriales.Weeksellaceae.Weeksellaceae_unclassified.Otu0002	recovery period	exposed	5.185	<0.0001
Proteobacteria.Gammaproteobacteria.Xanthomonadales	recovery period	exposed	3.148	0.0286
Proteobacteria.Gammaproteobacteria.Xanthomonadales.Xanthomonadaceae	recovery period	exposed	3.128	0.0286
Verrucomicrobia	recovery period	unexposed	5.264	<0.0001
Verrucomicrobia.Verrucomicrobiae	recovery period	unexposed	5.264	<0.0001
Verrucomicrobia.Verrucomicrobiae.Verrucomicrobiales	recovery period	unexposed	5.264	<0.0001
Verrucomicrobia.Verrucomicrobiae.Verrucomicrobiales.Rubritaleaceae	recovery period	unexposed	5.262	<0.0001
Verrucomicrobia.Verrucomicrobiae.Verrucomicrobiales.Rubritaleaceae.Rubritaleaceae_unclassified	recovery period	unexposed	5.262	<0.0001
Verrucomicrobia.Verrucomicrobiae.Verrucomicrobiales.Rubritaleaceae.Rubritaleaceae_unclassified.Otu0001	recovery period	unexposed	5.262	<0.0001
Verrucomicrobia.Verrucomicrobiae.Verrucomicrobiales.Verrucomicrobiales_unclassified	recovery period	unexposed	4.889	<0.0001
Verrucomicrobia.Verrucomicrobiae.Verrucomicrobiales.Verrucomicrobiales_unclassified.Verrucomicrobiales_unclassified	recovery period	unexposed	4.761	<0.0001
